# Rapid Pain Reduction and Wound Closure Following Treatment With an Amorphous Silica-Based Fiber Wound Matrix in Partial-Thickness Thermal Burns: A Case Report

**DOI:** 10.7759/cureus.116638

**Published:** 2026-09-21

**Authors:** Adam M Jorgensen, William L Hickerson

**Affiliations:** 1 Research and Development, SiOxMed, Winston Salem, USA

**Keywords:** amorphous silica, burn pain, burn wound healing, pain management, partial-thickness burns, re-epithelialization, silica-based fiber, thermal burns, wound matrix

## Abstract

Partial-thickness thermal burns can cause substantial pain and require effective wound management to support re-epithelialization. A 30-year-old man sustained 3%-4% total body surface area partial-thickness burns of the left hand, forearm, elbow, and abdomen following a gasoline-accelerated brush fire. Despite emergency debridement, dressings, and multimodal analgesia, pain remained 6-10/10. Approximately two days after injury, an amorphous silica-based fiber (SBF) wound matrix was applied to the open wounds, while a silica-based cream (SBC) was applied to surrounding intact skin. At initial SBF/SBC treatment, patient-reported pain decreased from 7/10 to 0/10 on a 0-10 numerical rating scale (NRS), with substantial relief reported within approximately 5-10 minutes. Across 12 paired assessments, mean pain decreased from 2.46/10 before treatment to 1.17/10 afterward. Serial planimetry demonstrated progressive wound closure with complete measured closure by Day 10, with sustained epithelial coverage and minimal scarring at Day 63. Because partial-thickness burns may spontaneously re-epithelialize, and this represents a single uncontrolled case with prior and concomitant treatment, causality cannot be established. The observed temporal pain response and wound-healing trajectory support prospective controlled evaluation using standardized pain, analgesic-use, epithelialization, functional, and safety endpoints.

## Introduction

Burn injuries compromise the skin barrier and can result in substantial pain, infection risk, impaired function, and scarring [1]. Partial-thickness burns may be particularly painful as viable nociceptive structures remain within injured dermal tissue, while wound cleansing, debridement, and dressing changes can further exacerbate pain [2]. Management therefore focuses on promoting re-epithelialization, protecting the wound, preserving function, and providing effective multimodal pain control [3].

Conventional management of partial-thickness burns includes wound cleansing, protective dressings, skin substitutes, maintenance of an appropriate wound environment, and multimodal analgesia [4]. Despite these measures, dressing changes and wound manipulation may remain painful. Amorphous silica-based wound matrices represent a distinct synthetic approach to wound management, but clinical experience with these materials in acute thermal burns remains limited.

We report the clinical course of a patient with multiple partial-thickness thermal burns initially managed with conventional emergency burn care and subsequently treated with an amorphous silica-based fiber wound matrix (SBF; SiOxD Wound Matrix, SiOxMed, Winston-Salem, NC, USA) in conjunction with a topical silica-based cream (SBC, SiOxC Cream, SiOxMed, Winston-Salem, NC, USA) applied to surrounding intact skin. This case describes the temporal relationship between treatment application and patient-reported pain, along with serial quantitative and photographic assessment of wound closure.

## Case presentation

A 30-year-old man with no significant medical history sustained a gasoline-accelerated thermal burn while igniting an outdoor brush pile. He presented to the emergency department within approximately one hour with severe burning and stabbing pain of the left hand and forearm. Examination demonstrated 3%-4% total body surface area partial-thickness burns involving the left palmar hand, forearm, elbow, and hemi-abdomen. The upper-extremity burns were non-circumferential, with preserved sensation, motor function, and perfusion. No inhalational injury or additional trauma was identified.

Initial management included intravenous fentanyl and ketamine, oral morphine, bedside debridement, and dressings with Mepilex Ag, Kerlix, and burn netting. Despite multimodal analgesia, documented pain scores ranged from 6/10 to 10/10. The patient was discharged with wound-care instructions and acetaminophen, ibuprofen, and short-term morphine.

Two days after discharge, the patient received SBF and SBC from SiOxMed for personal self-treatment. For clarity, Day 0 (D0) denotes initiation of SBF/SBC treatment, with subsequent treatment days reported relative to this time point; D-2 denotes the initial injury and emergency-treatment period. After receiving the materials, the patient voluntarily agreed to document and share his experience through a daily diary, patient-reported pain scores, and serial wound photographs. These patient-generated records, together with available clinical documentation, were subsequently retrospectively reviewed for this case report.

During self-treatment, dressing removal and wound manipulation produced substantial pain. SBF was applied directly to the open wound surfaces with light pressure to achieve full and even contact with the wound bed. SBC was applied only to surrounding intact skin and not to breached skin or the open wound bed. The products were used according to their instructions for use, with SBF reapplied to exposed wound areas as needed. Serial photographs demonstrated progressive epithelial coverage of the left forearm, palmar hand, and abdominal wounds. Figure 1 includes the initial SBF application at D0, subsequent wound appearance, visible epithelial coverage by Day 9, and sustained epithelial coverage at Day 63. No surgical grafting was documented, and no wound infection or serious treatment-associated adverse event was identified in the available follow-up records.

**Figure 1:**
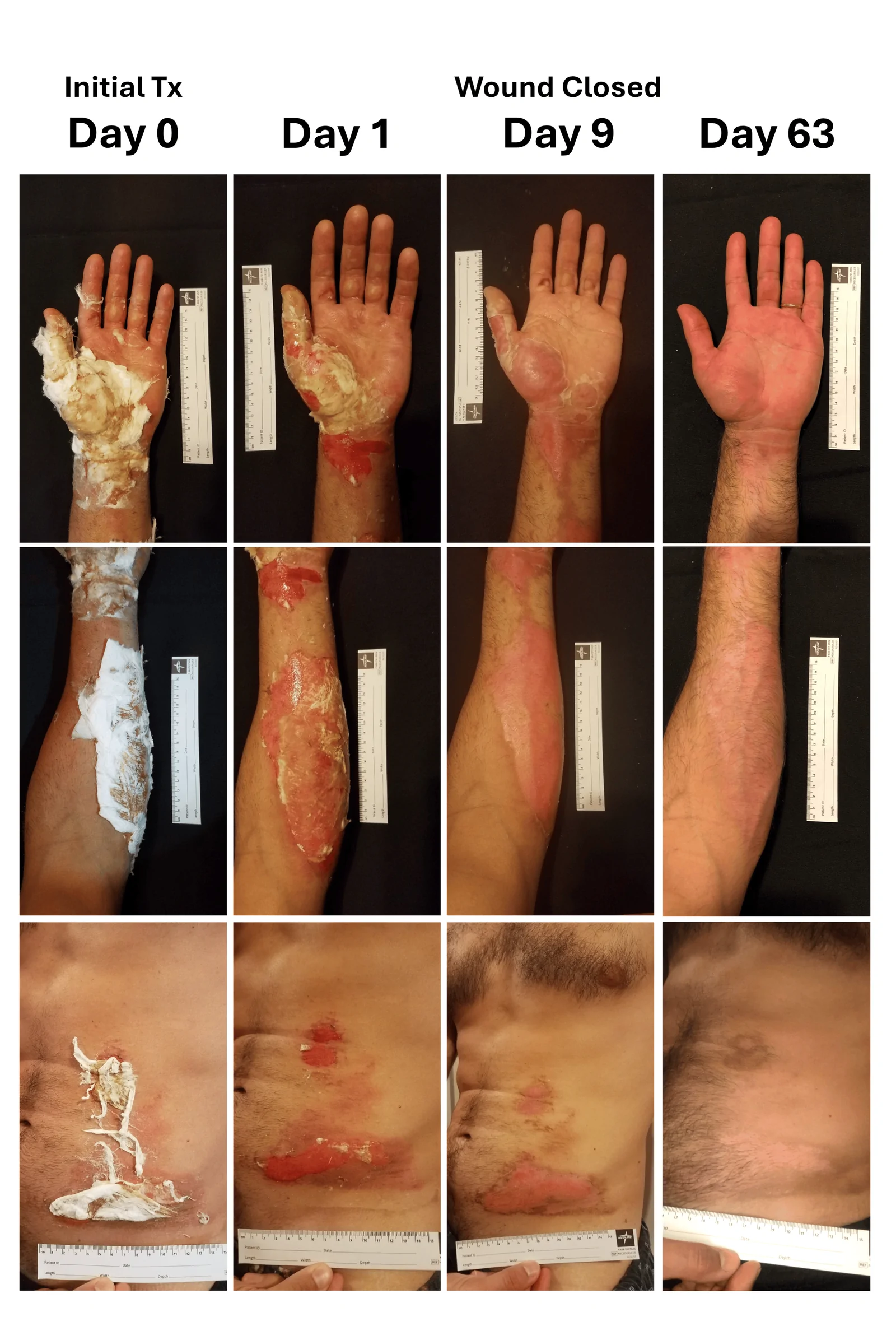
Serial appearance of partial-thickness thermal burns following initiation of amorphous silica-based fiber wound matrix treatment. Day 0 demonstrates the initial application of SBF to the open wounds of the left palmar hand, forearm, and abdomen. Subsequent images demonstrate progressive epithelialization, visible epithelial coverage by Day 9, and sustained epithelial coverage at Day 63.

Serial wound-area measurements were retrospectively derived from standardized digital photographs using quantitative two-dimensional planimetry according to Molnar et al. [5]. Images were spatially calibrated using a dimensional reference, wound margins were digitally traced, and open wound area was calculated in square centimeters. All measurements were performed by the same investigator. The combined open wound area on Day 1 was 225.40 cm^2^. Wound closure was calculated as 100 × (1 - (follow-up open wound area/initial open wound area)). Quantitative planimetry was discontinued after the measured open wound area reached 0 cm^2^; the Day 63 photographs document sustained epithelial coverage during longer-term follow-up rather than an additional quantitative wound-area measurement (Table 1).

**Table 1:** Serial quantitative wound closure. Treatment days are reported relative to the initiation of SBF/SBC treatment (D0). Measured wound closure was calculated relative to the initial quantitative wound-area measurement. SBF: silica-based fiber; SBC: silica-based cream

Treatment day	Combined open wound area (cm^2^)	Measured wound closure
1	225.402	0%
2	187.465	16.8%
4	111.087	50.7%
6	100.295	55.5%
7	13.444	94.0%
8	2.419	98.9%
9	0.240	99.9%
10	0	100%

Pain was patient-reported using a 0-10 numerical rating scale (NRS), with 0 representing no pain and 10 the worst pain. During the initial injury and emergency-treatment period (D−2), documented pain reached 10/10. At initiation of SBF/SBC treatment (D0), pain decreased from 7/10 to 0/10, with substantial relief reported within approximately 5-10 minutes. Similar rapid reductions were reported following treatment of the hand, forearm, elbow, and abdominal wounds. Pain intermittently recurred, particularly overnight and following wound manipulation or showering, and treatment was reapplied as needed. Pain assessments were patient-generated and were not collected under a prospectively standardized pain-assessment protocol; therefore, assessment intervals were not identical across time points. Across the subsequent 12 paired assessments from D1 through D12, mean NRS pain decreased from 2.46/10 before treatment to 1.17/10 afterward. Pain decreased in eight assessments and remained unchanged in four (Figure 2 and Table 2).

**Figure 2:**
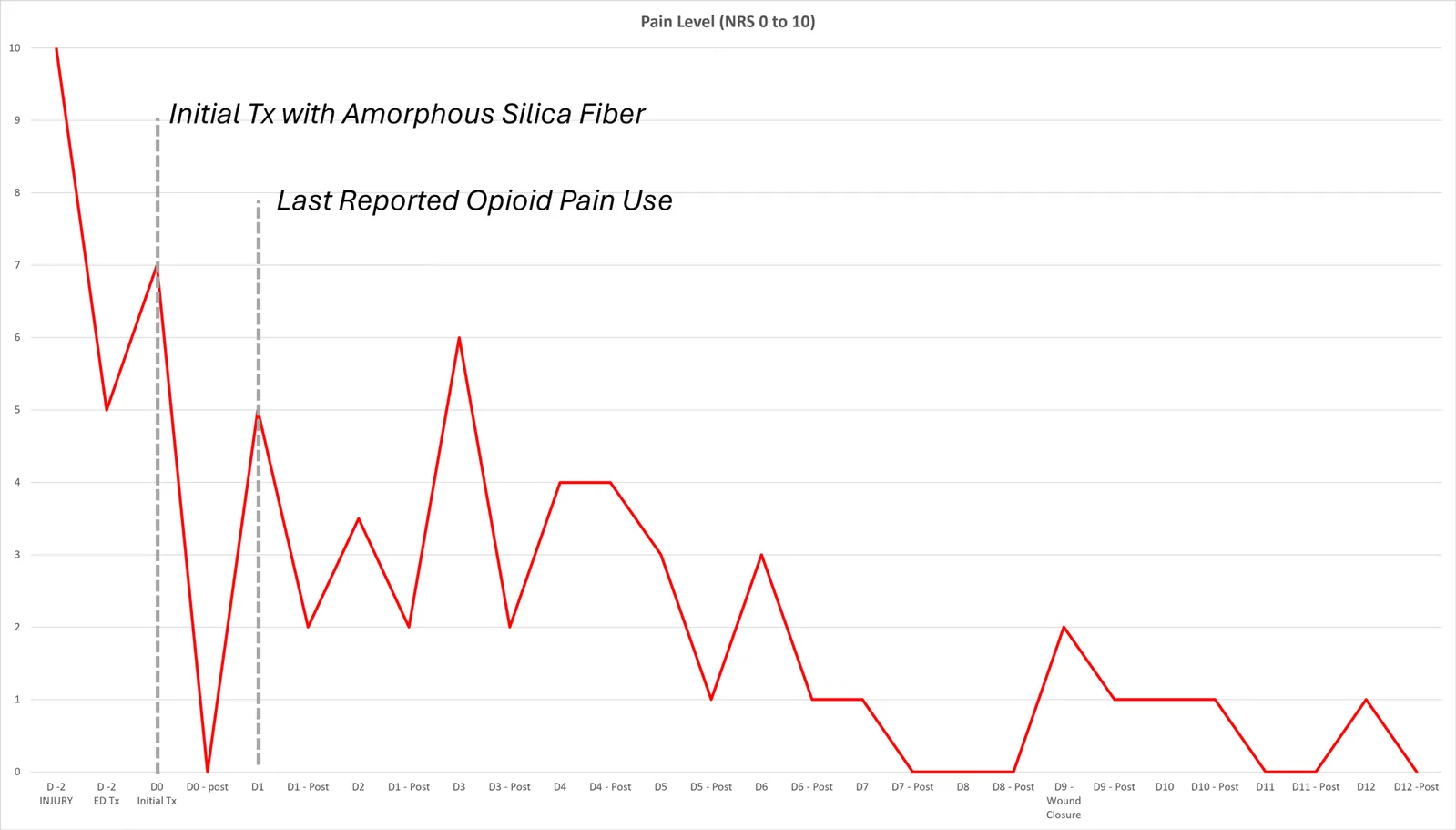
Patient-reported pain before and after initiation of SBF/SBC treatment. Pain was patient-reported using a 0-10 numerical rating scale (NRS). D-2 represents the injury and initial emergency treatment period, and D0 represents the initiation of SBF/SBC treatment. Subsequent paired pre- and post-treatment NRS scores are shown through D12. The timing of the last reported opioid analgesic use is indicated. SBF: silica-based fiber; SBC: silica-based cream

**Table 2:** Patient-reported pain before and after initiation of SBF/SBC treatment. D0 represents initiation of treatment with SBF and SBC; D-2 represents the initial injury and emergency treatment period. Pain was patient-reported using a 0-10 NRS. The D1-D12 observations comprise the 12 subsequent paired outpatient assessments used to calculate the reported mean pre- and post-treatment pain scores. SBF: silica-based fiber; SBC: silica-based cream; ED: emergency department

Treatment day	Assessment	Pre-treatment NRS pain	Post-treatment NRS pain	Change
D-2	Injury/ED treatment	10.0	5.0	-5.0
D0	Initial SBF/SBC treatment	7.0	0	-7.0
D1	SBF/SBC treatment	5.0	2.0	-3.0
D2	SBF/SBC treatment	3.5	2.0	-1.5
D3	SBF/SBC treatment	6.0	2.0	-4.0
D4	SBF/SBC treatment	4.0	4.0	0
D5	SBF/SBC treatment	3.0	1.0	-2.0
D6	SBF/SBC treatment	3.0	1.0	-2.0
D7	SBF/SBC treatment	1.0	0	-1.0
D8	SBF/SBC treatment	0	0	0
D9	SBF/SBC treatment	2.0	1.0	-1.0
D10	SBF/SBC treatment	1.0	1.0	0
D11	SBF/SBC treatment	0	0	0
D12	SBF/SBC treatment	1.0	0	-1.0

## Discussion

This case highlights two clinically relevant observations following outpatient treatment with SBF and SBC after conventional management of partial-thickness burns: a rapid temporal reduction in patient-reported pain following treatment and progressive wound closure.

The observed wound-healing trajectory is also consistent with emerging preclinical evidence supporting a favorable wound environment with SBF. In a porcine full-thickness burn model, SBF-treated wounds demonstrated reduced inflammatory cell infiltration, improved epidermal rete ridge formation, and more organized extracellular matrix collagen remodeling compared with untreated wounds, while cellular proliferation remained comparable between groups [6]. Clinical studies validating translation of these findings have not yet been performed. These preclinical findings therefore provide biological context for the progressive epithelialization observed in this case but do not establish the mechanism of the clinical response. Following application of SBF to the open wound bed and SBC to the surrounding intact skin, the patient reported pain reduction within approximately 5-10 minutes at multiple anatomical sites. The initial paired assessment decreased from 7/10 to 0/10, and eight of 12 subsequent paired assessments demonstrated lower post-treatment pain. Although the mechanism cannot be determined from this case, the repeated temporal relationship between treatment and pain reduction is clinically noteworthy. SBF may reduce mechanical and environmental stimulation of exposed wound surfaces while providing a protective interface that supports a moist wound environment.

No wound infection was documented during follow-up. However, this single uncontrolled observation does not permit conclusions regarding infection prevention or antimicrobial activity.

Strengths of this report include documented injury characteristics and initial treatment, repeated pain assessments, serial photography, and quantitative wound measurements independently. Limitations include the single uncontrolled design, prior debridement and silver-containing dressing therapy, concomitant systemic analgesia, concurrent administration of SBF and SBC, subjective and unblinded pain assessments, and absence of a prospective standardized treatment protocol. Pain was self-reported using a 0-10 NRS and was not assessed under a blinded or prospectively standardized protocol. Analgesic administration was not consistently time-locked to pain assessments. No opioid use was documented after D0; however, a complete medication diary was unavailable.

Importantly, partial-thickness burns may spontaneously re-epithelialize without grafting. The uncontrolled design of this case therefore does not permit determination of whether SBF/SBC accelerated wound closure relative to conventional wound care, grafting, or other treatment approaches. Similarly, the observed pain reductions cannot be attributed solely to SBF or SBC. The wound-healing trajectory and pain response should therefore be interpreted descriptively rather than as evidence of comparative efficacy.

Accordingly, these findings are hypothesis-generating. Prospective controlled studies are warranted to determine whether SBF and SBC contribute to pain reduction or wound healing and should incorporate standardized pain assessments, analgesic consumption, time to complete epithelialization, validated wound measurements, infection, functional recovery, scarring, and safety endpoints.

## Conclusions

This case demonstrates the clinical course of 3%-4% total body surface area partial-thickness thermal burns treated with conventional emergency care followed by application of an amorphous SBF wound matrix to the open wounds and SBC to the surrounding intact skin. Initiation of SBF/SBC treatment was temporally associated with rapid patient-reported pain reduction, including an initial decrease from NRS 7/10 to 0/10 within approximately 5-10 minutes. Serial planimetry demonstrated progressive wound closure, reaching 99.9% measured closure by Day 9 and complete measured closure by Day 10, with sustained epithelial coverage documented at Day 63. Although partial-thickness burns may spontaneously re-epithelialize and the uncontrolled design precludes establishing causality, the rapid and repeated temporal association between SBF/SBC treatment and pain reduction, together with the observed wound-closure trajectory, is clinically noteworthy. These findings support prospective controlled evaluation of SBF/SBC using standardized pain, analgesic-use, epithelialization, functional, and safety endpoints.
